# What works in appraisal meetings for newly graduated doctors? – and what doesn’t?

**DOI:** 10.1186/s12909-022-03357-z

**Published:** 2022-04-22

**Authors:** Marianne Kleis Møller, Anita Sørensen, Pernille Andreassen, Bente Malling

**Affiliations:** 1 Department of Quality and Education, Aarhus University Hospital, Olof Palmes Allé 13, stuen, DK-8200 Aarhus, Denmark; 2 Administration, Randers Regional Hospital, Skovlyvej 15, DK-8930 Randers, Denmark; 3 Centre for Health Sciences Education, Health, Aarhus University, Palle Juul-Jensens Boulevard 82, Bygn. A, DK-8200 Aarhus, Denmark; 4 Department of Clinical Medicine, Aarhus University, Palle Juul-Jensens Boulevard 82, Bygn. A, DK-8200 Aarhus, Denmark

**Keywords:** Appraisal meetings, Learning plans, Educational advisor, Faculty development, Postgraduate medical education, Advisory program

## Abstract

**Background:**

In Denmark a national formal advisory program (NFAP) is mandatory in Postgraduate Medical Education (PGME). According to this, an educational advisor is assigned to each doctor in every clinical rotation to guide and oversee the work and learning progress of the trainee.

This study explores why newly graduated trainees evaluated the appraisal meetings in the advisory program as either beneficial (successes) or not beneficial (non-successes).

**Methods:**

Inspired by the Success Case Method, a survey was conducted among all 129 doctors employed in their first six-month clinical rotation of postgraduate medical education (PGY1) in the Central Denmark Region. A cluster analysis resulted in a group with eight successes respectively seven non-successes. Semi-structured interviews were conducted with six successes and five non-successes.

**Results:**

In contrast to non-successes, the successes had longer appraisal meetings and their advisor introduced them to purpose and process of meetings including use of the personal learning plan. Successes received feedback on clinical skills, overall global performance and career plans. The successes perceived their advisors as prepared, skilled and motivated and the advisor acted as a contact person.

To the successes, the appraisal meetings fostered clarification of and reflections on educational goals, progress and career as well as self-confidence and a sense of security.

**Conclusion:**

Success with appraisal meetings seemed to depend on advisor’s skills and motivation including willingness to prioritize time for this task.

The results from this study indicate the importance of faculty development. It also raises the question if all doctors should serve as advisors or if this task should be assigned to the most motivated candidates.

**Supplementary Information:**

The online version contains supplementary material available at 10.1186/s12909-022-03357-z.

## Background

Ensuring the appropriate knowledge, skills and behavior of doctors in clinical training (trainees) is one of the keys to provide excellent care and ensure patient safety [1–4]. Medical authorities thus increasingly aim to provide standards for general and individual training exemplified by the standards issued by the National Health Authorities in the UK and Denmark [5, 6]. To ensure that these standards are met, an experienced doctor must guide and supervise the work and progress of each trainee in accordance with the specific learning objectives and milestones for each clinical rotation period as well as the entire specialist training program (postgraduate medical education) [3, 4, 7].

The organization of trainee supervision in postgraduate medical education (PGME) varies between countries ranging from voluntary mentor-mentee relations [8] to structured mandatory programs with appointed educational advisors [5, 6]. Previous qualitative studies have described that successful mentor-mentee relations in academic medicine are characterized by reciprocity, mutual respect, clear expectations and personal relations [9, 10]. A literature review focusing on mentoring in emergency medicine recommended clarification of expectations of both mentor and mentee, structure with regular meetings and identification of the mentee’s short and long-term goals [11]. The mentor’s ability to act as an active listener, identify potential strengths in the mentee and assist the mentee in defining and reaching goals facilitates good mentoring relationships [12]. Among residents, attention to personal development and mentors reporting on mentee progress was associated with an overall satisfaction with the mentorship [13]. A more recent thematic review found that postgraduate mentees request their mentors to have professional experience and network connections and also be able to provide research-related, professional and emotional support [14].

**Fig. 1:**
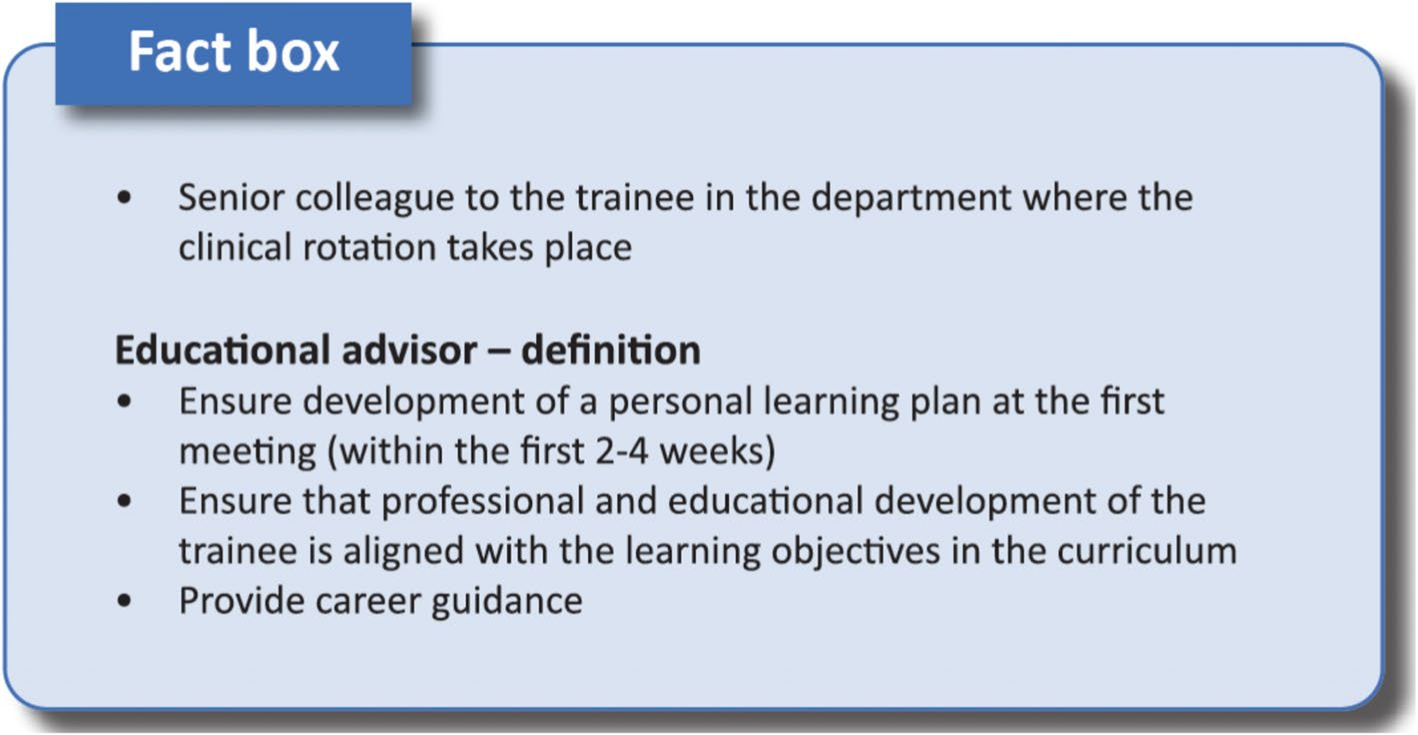
The definition and the tasks of the educational advisor (EA)

In Denmark, as well as in the UK, a national formal advisor program (NFAP) is a mandatory part of postgraduate medical education [15, 16]. The purpose of the Danish NFAP is to ensure professional development in accordance with learning objectives in the curriculum. In this program, a designated educational advisor (EA) is appointed to every trainee in each clinical rotation period. The definition and the tasks of the EA are similar to the Educational Supervisor in the UK National Health Services (NHS) [17] and are listed in the Fig. 1.

In 2018, we published the results from a questionnaire study on implementation of the NFAP among trainees employed in their first six-month clinical rotation of the PGME (PGY1 doctors) in Central Denmark Region. We found that overall appraisal meetings and learning plans were well implemented and supported the development of clinical competences, although learning plans were developed with delay, lacked plans for assessment and were not regularly adjusted [18]. The study showed that the appraisal meetings and the development of a learning plan influenced the benefits and value of the NFAP the most (40% each), while the coherence between the NFAS and the daily clinical work only contributed to the benefits with half the strength (20%) of the appraisal meetings and the learning plan respectively. (Sørensen et al. DMJ). The results called for a more thorough investigation as to which elements of the appraisal meeting were most beneficial for the PGY1 doctors in order to define areas where the appraisal system could be improved, since appraisal meetings are central to many advisory programs throughout the world.

The aim of the current study was to explore PGY1 doctors perception of appraisal meetings as part of a structured advisory program in order to identify differences between the PGY1 doctors who evaluated the appraisal meetings most (success) and least (non-success) beneficial.

## Methods

The design of this study was inspired by the Success Case Method (SCM), which is a research-based mixed methods approach to explore the effect of an organizational initiative [19]. The method is based on the idea that we learn best from those individuals who have been most successful and least successful respectively in applying new learning in their work [20]. Thus the SCM prescribes an analysis and comparison between participants with most success and participants with least success. In our study success/non-success was defined as the PGY1 doctors’ perception of usefulness of the appraisal meetings.

The SCM consists of two steps: A survey identifying the most likely successes and non-successes followed by individual interviews with about a handful of representatives from the potential successes and non-successes to uncover major differences between success and non-success with a specific initiative [19].

### Step 1: survey

A survey was conducted among all 129 PGY1 doctors in their first clinical rotation in Central Denmark Region.

The questionnaire was developed by the researchers and constructed to match the rules and recommendations by the Danish Ministry of Health regarding NFAP. The development of the questionnaire and main findings from the survey have been described previously [18]. A cluster analysis was performed to identify the participants with most and least success respectively.

Of 115 actively employed doctors, 67% (77/115) completed more than half the questions and were included in the cluster analysis. The cluster analysis used in this study was modified as to reflect the methodology of the SMC. All possible number of clusters in the range from 2 to 20 clusters was manually investigated. The best separations were seen with 11 clusters and 8 respondents were identified as successes and 7 respondents were identified as non-successes.

### Step 2: interviews

The 15 PGY1 doctors identified as successes and non-successes, respectively, were invited to participate in an interview by mail. In total 11 semi-structured interviews were conducted, in all including six successes and five non-successes. The interview guide was developed in accordance with the SCM, and included questions addressing the appraisal meetings and the personal learning plan [19]. The questions posed were: “What was used? (how, when and where)”, “What results were achieved? (what was different)”, “What good did it do? (value of result)”, “What helped?” and “What were the barriers?” The interview guide is provided in Additional file 1: Appendix 1 (in Danish) and Additional file 2: Appendix 2 (translated).

The experienced interviewer as well as the participating PGY1 doctors were blinded to the category of the success or non-success prior to the interviews to reduce the risk of bias and ensure an explorative approach.

Two interviews were carried out face-to-face after the interviewer had observed an appraisal meeting; the rest of the interviews were conducted by telephone. The interviews lasted between 25 and 35 min and were audio-recorded and transcribed verbatim.

Transcriptions were read through several times and subsequently analyzed following the qualitative content analysis approach [21] by the authors PA and AS. First, initial codes were generated, discussed and further explored in relation to the full data set. Secondly, memos were written and discussed among all four authors before agreeing on main themes. Finally, findings were classified according to the SCM categories.

## Results

The interviews revealed several differences between the interviewed six PGY1 doctors with potential success and the five PGY1 doctors with potential non-success. The content analysis resulted in the identification of six main themes. In the following the results are described according to the SCM categories. In the Table 1 the results are summarized and illustrated by quotations from the PGY1 doctors.

**Table 1:**
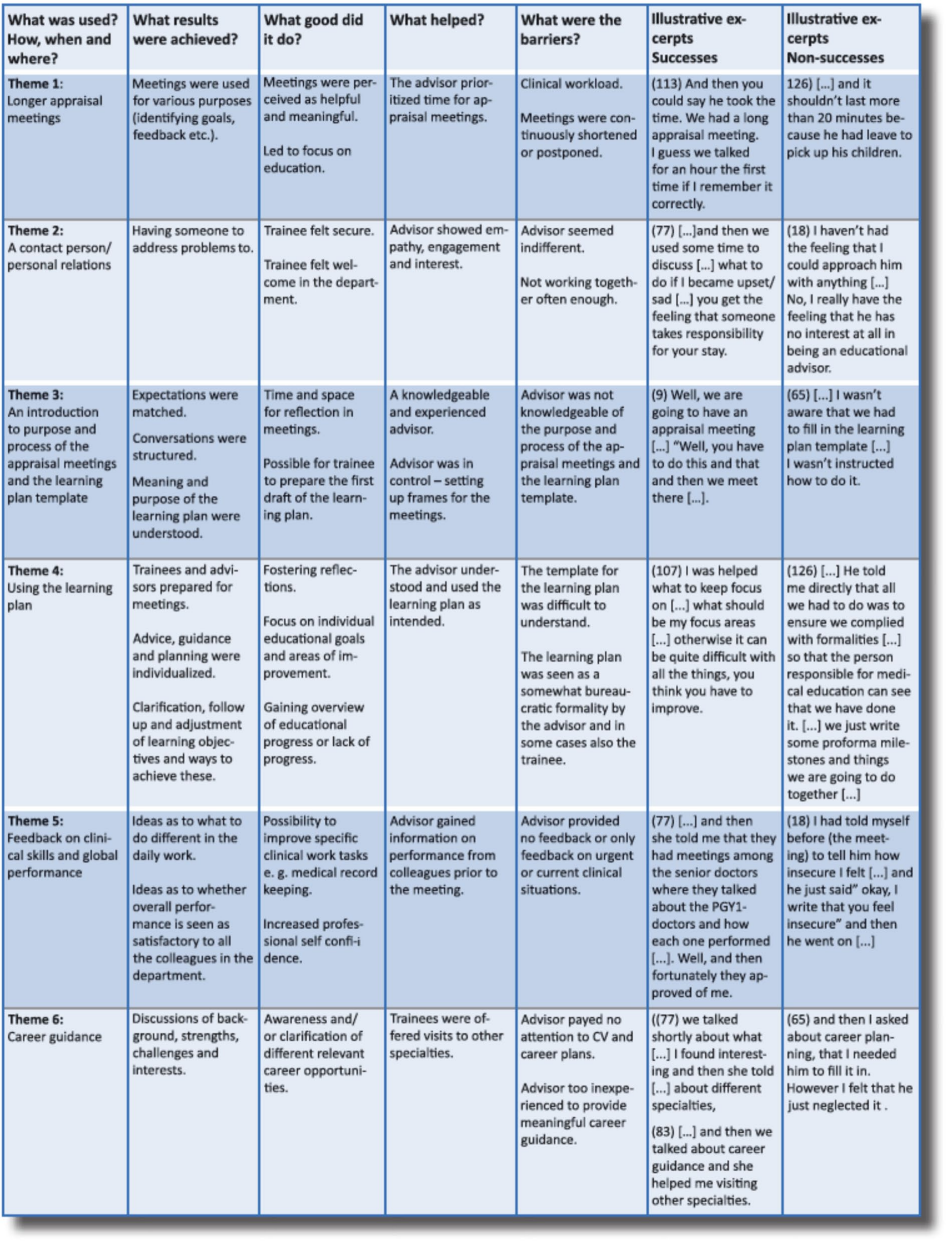
Main findings on content, consequences, facilitators and barriers to the appraisal meetings organized according to the SCM categories and the themes emerging in the analysis

### What was used?

There were clear differences between the length of the appraisal meetings. We found that successes had appraisal meetings lasting approximately 60 min, whereas the non-successes described their meetings as short and rushed - sometimes as short as 10 min. The successes experienced a clear introduction to the purpose of the meetings. Also, successes were introduced to the learning plan template and expected to use it. Furthermore, the successes, in contrast to the non-successes, were offered career guidance and received feedback on overall global as well as clinical skills performance. Overall, the successes perceived their EA as an approachable contact person.

### What results were achieved?

The successes expressed the importance of having a personal relation to the EA and someone to address problems to. Introduction to the purpose and process of appraisal meetings facilitated structured conversations and matching of expectations. The PGY1 doctors in the success group stated that using the learning plan helped them and their EA to be prepared for the meetings and also contributed to clarification, follow up and adjustment of learning objectives. In this way, the learning plan became an effective tool for successes to take control of their education in a busy clinical setting. Compared to the non-successes, the successes experienced a more individualized conversation with their EA including ideas on what to do different in the daily work as well as discussions of background, strengths, challenges and interests.

### What good did it do?

Overall, the six successes experienced the appraisal meetings as meaningful and helpful, and used them to gain overview of the purpose and goals of their current clinical rotation. Moreover, it helped them to keep track of both their progress, and areas where they needed to improve. In this sense, the meetings both helped them reach goals and set new milestones as well as reflect on their learning. The perception of a genuine interest from the EA in the PGY1 doctor as a person made the successes feel more welcome at the department. Feedback on clinical skills lead to increased professional confidence and discussions on background, interests etc. and lead to clarification of relevant career options. The non-successes received feedback on ongoing clinical problems but not on their general professional development.

### What helped?

Knowledge, attitude and experience of the EA were themes for perceived success. Deep insight in the NFAP and the use of the personal learning plan as a tool to facilitate the professional development was experienced as very helpful for the successes. Frames for the appraisal meetings, a clear agenda and prioritization of time were important factors. The ability of the EA to show empathy, engagement and interest for the PGY1 doctor contributed to the perception of the EA being a contact person. The EAs of successes also included information from colleagues in their feedback to the trainee’s overall global performance.

### What were the barriers?

The EAs of the non-successes were perceived as indifferent to the PGY1 doctors and to the task of being an EA. There was a lack of knowledge of the purpose and process of the appraisal meetings and of how to use the personal learning plan. Clinical workload was perceived as a barrier and appraisal meetings were often cancelled with short notice. Another difference was the experience of non-successes of not getting any feedback on their overall clinical performance or only feedback on urgent or current situations. The non-successes did not receive any career guidance – some even experienced ignorance when trying to bring up a carrier issue.

The non-successes expressed that their EA regarded appraisal meetings and the personal learning plan as a bureaucratic formality without any value.

## Discussion

In this study, the SMC was used as an inspiration to distinguish participants who experienced the most and the least success of the appraisal meetings as a part of a mandatory NFAP. We found that the perception of success was associated with the duration of appraisal meetings, the relationship with the EA, introduction to the purpose of appraisal meetings, guidance in development and use of personal learning plans, feedback on performance as well as career guidance. Each theme is discussed below.

### Longer appraisal meetings

Our study demonstrated that the duration of the meetings was longer for successes than non-successes. Both successes and non-successes described lack of time as a barrier to appraisal meetings. In the perception of the successes the advisors managed to allocate sufficient time and thereby demonstrated willingness to prioritize time for appraisal meetings.

There is no consensus on frequency or duration of meetings in mentoring relationships [9, 14], although lack of time is a well described barrier to effective mentoring [9–11, 22–25]. The latter corresponds with our result that too short and rushed meetings had a negative impact on trainees’ perceived benefits of the NFAP. Several authors have suggested scheduled meetings, protected time or time allocated in the job plan [24, 25] as in Denmark [15] and UK [17]. However, this does not necessary circumvent the issues with lack of time, as even allocated time can be impinged by the clinical workload pressure [24].

Even though our results showed a positive relation between time used and satisfaction with appraisal meetings, it did not prove causality. However, meetings can probably be too short to make a positive difference for the guiding of trainees’ work and progress. This suggests enrolling only senior staff prepared to allocate time to manage the role of the EA [26].

### A contact person – personal relations

The success PGY1 doctors in this study perceived their EA as approachable, dedicated and engaged. Previous studies of mentoring in medical education have found that mentors should ideally have seniority, be approachable and accessible, understanding, patient, dedicated, responsive as well as active listeners [8, 11, 12, 22, 25, 27]. Effective mentors should proactively check in with mentees to see how they are doing [9]. Some stressed the importance of selecting motivated and engaged mentors, ensuring that these have an interest in professional development in the workplace [28]. Finally, perceived closeness to the mentor at work seems important to the mentee [8, 26].

The present study supports the importance of appointing only motivated doctors to the role as EAs.

### An introduction to purpose and process

In the case of the successes, clarification of the purpose and process of the appraisal meetings lead to more structured conversations, leaving time and space for reflection. In contrast, the non-successes reported no introduction to the meetings. This might have had a negative impact on the perception of non-successes of the appraisal meetings, since clarifying mutual expectations at the beginning of a mentoring relationship are found critical to building effective mentoring relationships [9, 11, 14, 26]. Furthermore, it points out the importance of faculty development to supply advisors with proper knowledge of the advisory system and skills on how to use learning plans and give feedback [9, 22, 29]. In this way the organization demonstrates value of learning in the workplace [30] and recognizes the role that faculty development plays in curricular changes and development [22, 28, 31].

The present study supports the relevance of faculty development to achieve success in mentoring or advisory programs. It would be relevant to study if faculty development contributes to increase the motivation to act as mentors and EAs.

### Help in using the learning plan

The successes were guided in the development and use of the learning plans, which worked as a tool to initiate reflections before appraisal meetings, and as a basis for the conversation with the advisor. To the successes, the learning plan became an effective tool to take control of their education in a busy clinical setting. However, if the learning plan was regarded a formality by the advisor and / or the trainee, or if neither of them understood or acknowledged its role as a tool, the learning plan became a demotivating factor as was the case for the non-successes.

Individualized learning plans are supposed to help improve development of self-directed, lifelong learning by actively engaging learners to take ownership of their own learning [32–34]. Individual learning plans may serve as a checklist to frame the mentor’s meetings with the mentee [9]. However, trainees need help from seniors to develop and use their learning plans [16, 26, 32].

In order to be helpful, a learning plan must probably be revisited on a regular basis. This must be encouraged, since, according to Su-Ting et al. around half of the trainees did not remember to work on their learning goals on a regular basis [33].

Thus, data from the present study support the relevance of a personal learning plan as a tool to support professional development, but also the importance of guidance and encouragement from the EA to use the plans.

Feedback on both clinical skills and overall global performance from the perspective of the PGY1 doctors was a valued part of the appraisal meetings for all participants in our study. However, some find that feedback on clinical performance should take place in the daily clinical work [35]. It therefore has been suggested to make a clear distinction between educational and clinical supervision [26]. Educational supervision involves support of the individual trainee with agenda-setting and planning in the context of a training program. Moreover, it should incorporate overview of the progress in the light of independent assessments of the trainee’s clinical performance, carried out by clinical supervisors [26]. Clinical supervision, on the other hand, draws on the training agenda agreed upon with the educational supervisor to identify and support the training required [26].

This suggests that appraisal meetings should merely focus on global performance, passing millstones, as well as setting new goals for professional and personal development, which is actually the intension with the appraisal meetings [6]. This emphasizes the need for, faculty to discuss the purpose of the program as well as the tasks of an EA to ensure the distinction between clinical and educational supervision. This might lead to a more fruitful feedback on overall performance in the appraisal meetings.

### Career guidance

Career guidance is a mandatory part of appraisal meetings. To the successes, the inclusion of career guidance in the appraisal meetings contributed to reflections on relevant career choices on the background of discussions of e.g. strengths and interests. The non-successes reported receiving little or no career guidance.

Mentoring is important to career progression [13, 36], and it is relevant to combine advisors’ feedback on progression of clinical performance with career guidance, as trainees’ strengths and weaknesses should be essential to reflections on choice of career [9, 29].

Based on the results of the present study, career guidance should be carried out by the EA as he/she ideally has an overview of the competences of trainees and through the appraisal meetings the EA has the opportunity to discuss relevant career options on the background of the trainee’s strengths and weaknesses. It is possible that PGY1 doctors with no or insufficient career guidance may experience problems in their career progress. However, further studies are needed to explore the influence of lack of career guidance on future career.

### Limitations and perspectives for future research

It was a limitation in our study that only the PGY1 doctors were interviewed. Thus, the results presented here represent only their perspective. As success in appraisal meetings presumably depends on both the EA and the PGY1 and the relation they are able to build, it would be interesting to explore the perspective of the EAs and to observe the interaction between advisors and trainees.

The NHS and the NFAP represents organizational systems with defined roles of the EA and combined with a structure for regular meetings with the trainee. However, appraisal meetings probably are a central and important part of postgraduate medical education whether or not you have a nationally defined system. The results of the study thus seem to be relevant and usable in other countries and other contexts. We have chosen to discuss the results from the present study with results from studies on factors important to success or failure of mentoring, since most studies focus on mentor-mentee relations. In this light, our findings seem representative, since the same themes and barriers emerged in our study compared to studies on mentoring.

The majority of interviews were relatively short telephone interviews, and thus there was only limited time to build a relationship with the informants. Furthermore, the interviewer had limited familiarity with the NFAP at the beginning of the study. Thus, themes that emerged during the first interviews might have been more deeply explored. Continuing sampling until saturation of themes was not intended as the sample size was given by the cluster analysis in accordance with the SCM. However, we found a substantial agreement between the themes of importance to successes and non-successes, respectively. All authors participated in the data analysis, discussion of themes and sub-themes, thus increasing the dependability of the study.

## Conclusion

To be successful, in the perspective of PGY1 doctors, appraisal meetings should be of appropriate length and the purpose and process of both the meetings and the personal learning plan should be introduced. The EA should function as a contact person and the meetings should include feedback on global performance, professional development and progress as well as career guidance.

Appraisal meetings can provide clarification of and reflections on educational goals, progress and career as well as self-confidence and a sense of security for trainees, but success depends on advisor’s skills, motivation and prioritization. This indicates the importance of faculty development to take on the role as educational advisor, but it also raises the question if all doctors should serve as advisors or if this role should be assigned to the most motivated candidates.

## Supplementary Information

**Additional file 1.**

**Additional file 2.**

## Data Availability

The datasets generated and analyzed during the current study are not publicly available due to the risk of compromising individual privacy, but are available from the corresponding author on reasonable request.

## Abbreviations

EA: A designated educational advisor
NFAP: A national formal advisor program
PGY1 doctors: Trainees in their first six-month clinical rotation of postgraduate medical education
SCM: The Success Case Method
PGME: post graduate medical education

## References

[1] Bleakley A, Bligh J, Browne J (2011). Medical education for the future. Identity, power and location.

[2] Farnan JM, Petty LA, Georgitis E, Martin S, Chiu E, Prochaska M, Arora VM (2012). A systematic review: the effect of clinical supervision on patient and residency education outcomes. Acad Med.

[3] Forrest CN. Essential Guide to educational supervision in postgraduate medical education: Blackwell Publishing Ltd; 2009. isbn:978-1-405-17071-0.

[4] National Health Services, UK: Health Education England: SupervisionReport_FINAL1.pdf (hee.nhs.uk). retrived 211201.

[5] National Health Service, UK. Enhancing supervision for postgraduate doctors in training | Health Education England (hee.nhs.uk). retrived 211201.

[6] Danish National Board of Health (1998). The Danish National Board of Health. (1998) Guidance and evaluation in the training of specialists in Denmark.

[7] Kilminster S, Cottrel D, Grant J, Jolly B (2007). AMEE Guide no. 27: effective educational and clinical supervision. Med Teach.

[8] Flint JH, Jahangir AA, Browner BD, Mehta S (2009). The value of mentorship in orthopedic surgery resident education: the residents' perspective. J Bone Joint Surg Am.

[9] Straus SE, Johnson MO, Marquez C, Feldman MD (2013). Characteristics of successful and failed mentoring relationships: a qualitative study across two academic health centers. Acad Med.

[10] Ogdie A, Sparks JA, Angeles-Han ST, Bush K, Castelino FV, Golding A, Jiang Y, Kahlenberg JM, Kim AHJ, Lee YC, Machireddy K, Ombrello MJ, Shah AA, Wallace ZS, Nigrovic PA, Makris UE (2018). Barriers and facilitators of mentoring for trainees and early career investigators in rheumatology research: current state, identification of needs, and road map to an inter-institutional adult rheumatology mentoring program. Arthri Care Res (Hoboken).

[11] Yeung M, Nuth J, Stiell IG (2010). Mentoring in emergency medicine: the art and the evidence. CJEM.

[12] Williams LL (2004). The good-enough mentoring relationship (academic). Psychiatry.

[13] Ramanan RA, Taylor WC, Davis RB, Phillips RS (2006). Mentoring and career preparation in internal medicine residency training. J Gen Intern Med.

[14] Sng JH, Pei Y, Toh YP, Peh TY, Neo SH, Krishna LKR (2017). Mentoring relationships between senior physicians and junior doctors and/or medical students: a thematic review. Med Teach.

[15] Danish Ministry of Health. Recommendations no 9586 of 14/07/2008: Vejledning om kompetencevurdering i den lægelige videreuddannelse.( Recommendations on assesment in postgraduate medical education) 2008: in Danish.

[16] Danish Ministry of Health Executive order no 1257 of 25/10/2007: Executive order on the training of medical specialists 2007: in Danish.

[17] National Association og Clinical Tutors, UK: . Final Appendix 2 - Roles of Supervisors.pdf (onerm.dk) retrived 211201.

[18] Sørensen A, Møller MK, Andreassen P, Malling B. A SWOT analysis of how the youngest doctors perceive the formal Danish educational advisory program. Dan Med J. 2018;65(9):A5498. 30187859

[19] Brinkerhoff RO. The Success case Method: Find out quickly what's working and what´s not Berrett-Koehler Publishers. San Francisco. 2009. p. 1–25.

[20] Barrington G. The Success Case Method. Poster may 19, 2004 at Canadian Evaluation Society: https://evaluationcanada.ca/distribution/20040519_barrington_gail.pdf. retrived 211201.

[21] Emmerson RM, Fretz RI, Shaw LL. Writing Ethnographic Fieldnotes: University of Chicago Press; 1995.

[22] Straus SE, Chatur F, Taylor M (2009). Issues in the mentor-mentee relationship in academic medicine: a qualitative study. Acad Med.

[23] Kashiwagi DT, Varkey P, Cook DA (2013). Mentoring programs for physicians in academic medicine: a systematic review. Acad Med.

[24] Webb J, Brightwell A, Sarkar P, Rabbie R, Chakravorty I (2015). Peer mentoring for core medical trainees: uptake and impact. Postgrad Med J.

[25] Sambunjak D, Straus SE, Marusic A (2009). A systematic review of qualitative research on the meaning and characteristics of mentoring in academic medicine. J Gen Intern Med.

[26] Mellon A, Murdoch-Eaton D (2015). Supervisor or mentor: is there a difference? Implications for pediatric practice. Arch Dis Child.

[27] Woods SK, Burgess L, Kaminetzky C, McNeill D, Pinheiro S, Heflin MT (2010). Defining the roles of advisors and mentors in postgraduate medical education: faculty perceptions, roles, responsibilities, and resource needs. J Grad Med Educ.

[28] Billett S (2002). Toward a workplace pedagogy: guidance, participation and engagement. Adult Educ Q.

[29] Ssemata AS, Gladding S, John CC, Kiguli S (2017). Developing mentorship in a resource-limited context: a qualitative research study of the experiences and perceptions of the Makerere university student and faculty mentorship programme. BMC Med Educ.

[30] Steinert Y, Steinert Y (2014). Faculty development in the health professions. A focus on research and practice.

[31] Steinert Y (2012). Perspectives on faculty development 6/6 by 2020. Perspect Med Educ.

[32] Challis M (2000). AMEE medical education guide no. 19: personal learning plans. Med Teach.

[33] Su-Ting LT, Paterniti DA, Co JPT, West DC (2010). Successful Self-Directed Lifelong Learning in Medicine: A Conceptual Model Derived From Qualitative Analysis of a National Survey of Pediatric Residents. Acad Med.

[34] van Houten-Schat MA, Berkhout JJ, van Dijk N, Endedijk MD, Jaarsma ADC, Diemers AD (2018). Self-regulated learning in the clinical context: a systematic review. Med Educ.

[35] Watling CJ, Lingard L (2012). Toward meaningful evaluation of medical trainees: the influence of participants’ perceptions of the process. Adv Health Sci Educ.

[36] Sambunjak D (2015). Understanding wider environmental influences on mentoring: towards an ecological model of mentoring in academic medicine. Acta Med Acad.

[37] National Committee on Health Research Ethics, Denmark 2011: Act on Research Ethics Review of Health Research Projects | National Committee on Health Research Ethics – nvk.dk. retrived 211201.

