# Supplementary material for: What works in appraisal meetings for newly graduated doctors? – and what doesn’t?
Source: BMC Med Educ. 2022 Apr 22;22:306. doi: 10.1186/s12909-022-03357-z (PMC9027894; doi:10.1186/s12909-022-03357-z)
Supplement: Supplementary file 1 — Additional file 1. [file 12909_2022_3357_MOESM1_ESM.docx]

**Interviewguide SCM**

**April 2017, aus**

I modsætning til SCM så stilles alle spørgsmålene her til alle informanter, fordi intervieweren er blindet for hvem der er potentielle successer hhv. potentielle non-successer.

| **What was used? How, when and where?** | **What results were achived? What is different?** | **What good did it do (value)?** | **What helped?** | **What was the barriers?** | **Suggestions?** |
| --- | --- | --- | --- | --- | --- |
| 1A. Hvilke elementer fra vej-ledningen anvender du? Hvordan, hvor og hvornår anvender du dem? Hvilke objektive tegn er der herpå? | 2A. Hvad får vejledningen dig til at gøre anderledes i dit daglige arbejde?  Hvilke resultater medfører det? I hvilke situationer og hvordan kan det observeres? | 3A. Hvad bidrager vejledningen med/til?  I hvilke situationer og hvordan kan det observeres? | 4A. Hvilke faktorer har bidraget til dit udbytte af vejledningen? Hvad har du selv gjort? Hvad har andre gjort? Hvilke omstændigheder har haft betydning for dit udbytte? | 5A. Hvilke forhindringer er der i forhold til at få udbytte af vejledningen? | 6A. Hvad kunne gøre udbyttet af vejledningen større? |
| 1B. Hvilke elementer fra vejledningen anvender du ikke og hvorfor? |  | 3B. Hvilke uhensigtsmæssig-heder bidrager vejledningen til at forhindre/begrænse?  I hvilke situationer og hvordan kan det observeres? |  | 5B. Hvilke faktorer har konkret nedsat dit udbytte af vejledningen? |  |
|  |  |  |  |  |  |
