# Supplementary material for: What works in appraisal meetings for newly graduated doctors? – and what doesn’t?
Source: BMC Med Educ. 2022 Apr 22;22:306. doi: 10.1186/s12909-022-03357-z (PMC9027894; doi:10.1186/s12909-022-03357-z)
Supplement: Supplementary file 2 — Additional file 2. [file 12909_2022_3357_MOESM2_ESM.docx]

**Interviewguide PGY1-doctors**

**January 2022, MKM**

In contrast to SCM all questions are adressed to all informants, because the interviewer is blinded in regard to who is the potential success in contrast to the potential non-success.

| **What was used? How, when and where?** | **What results were achived? What is different?** | **What good did it do (value)?** | **What helped?** | **What were the barriers?** | **Suggestions?** |
| --- | --- | --- | --- | --- | --- |
| 1A. Which elements from the appraisal meetings do you use. How, were and when do you use it? Which objective signs can be seen? | 2A. How do appraisal meetings make you do things differently in your daily work. What are the results? In which situations and how can it be observed? | 3A. What do appraisal meetings give you in return? In which situations do you benefit and how can it be observed? | 4A. Which factors has contributed to your benefit of the appraisal meetings? What have you done yourself? What have others done? which circumstances have had influence on your profit? | 5A. Which obstacles were there in relation to use your profit/benefit from the appraisal meetings? | 6A. What could make the profit of the appraisal meetings better? |
| 1B. Which elements from the appraisal meetings do you not use and Why not?. |  |  |  | 5B. Which concrete factors have reduced your profit from the appraisal meetings? |  |
|  |  |  |  |  |  |
